# Hand Exoskeleton Design and Human–Machine Interaction Strategies for Rehabilitation

**DOI:** 10.3390/bioengineering9110682

**Published:** 2022-11-11

**Authors:** Kang Xia, Xianglei Chen, Xuedong Chang, Chongshuai Liu, Liwei Guo, Xiaobin Xu, Fangrui Lv, Yimin Wang, Han Sun, Jianfang Zhou

**Affiliations:** 1 College of Mechanical & Electrical Engineering, HoHai University, Nanjing 210098, China; 2 School of Mechanical, Medical and Process Engineering, Queensland University of Technology (QUT), Brisbane, QLD 4001, Australia; 3 Articular Orthopaedics, The Third Affiliated Hospital of Soochow University, Changzhou 213003, China

**Keywords:** hand exoskeleton design, motion simulation, rehabilitation, intention recognition, machine learning, deep learning

## Abstract

Stroke and related complications such as hemiplegia and disability create huge burdens for human society in the 21st century, which leads to a great need for rehabilitation and daily life assistance. To address this issue, continuous efforts are devoted in human–machine interaction (HMI) technology, which aims to capture and recognize users’ intentions and fulfil their needs via physical response. Based on the physiological structure of the human hand, a dimension-adjustable linkage-driven hand exoskeleton with 10 active degrees of freedom (DoFs) and 3 passive DoFs is proposed in this study, which grants high-level synergy with the human hand. Considering the weight of the adopted linkage design, the hand exoskeleton can be mounted on the existing up-limb exoskeleton system, which greatly diminishes the burden for users. Three rehabilitation/daily life assistance modes are developed (namely, robot-in-charge, therapist-in-charge, and patient-in-charge modes) to meet specific personal needs. To realize HMI, a thin-film force sensor matrix and Inertial Measurement Units (IMUs) are installed in both the hand exoskeleton and the corresponding controller. Outstanding sensor–machine synergy is confirmed by trigger rate evaluation, Kernel Density Estimation (KDE), and a confusion matrix. To recognize user intention, a genetic algorithm (GA) is applied to search for the optimal hyperparameters of a 1D Convolutional Neural Network (CNN), and the average intention-recognition accuracy for the eight actions/gestures examined reaches 97.1% (based on K-fold cross-validation). The hand exoskeleton system provides the possibility for people with limited exercise ability to conduct self-rehabilitation and complex daily activities.

## 1. Introduction

In the 21st century, the aged population has increased dramatically. Among elders, a considerable number of people suffer from stroke and related complications such as hemiplegia, disability, etc., which lead to problems in daily caring [1]. To restore self-care capabilities, stroke patients usually require a long rehabilitation period after surgery [2,3]. Patients’ needs at different rehabilitation stages vary, thus rehabilitation therapy should also be changed accordingly. To address this issue, human–machine interaction (HMI) technology is developed for rehabilitation exoskeletons [4,5,6]. In brief, all HMI technologies serve three purposes, which are intention capture, intention recognition, and physical response [7].

Capturing exoskeleton user intention traditionally relies on feedback from sensors, such as force transducers [8,9,10], cameras [11], strain gauges [12], and lasers [13], each of which possesses inadequate sensor–machine synergy in dealing with complex gesture/action and leads to low intention-recognition accuracy. Recently, electromyography (EMG) and electroencephalogram (EEG) have been extensively studied for HMI due to their high intention-detection accuracy potential, which benefits from multiple signal channels [14,15,16]. However, EMG and EEG usually require huge data manipulation efforts, which lead to a significant delay in real-time control [17]. To balance sensor–machine synergy and real-time control performance, sensor matrices have been developed in many studies. Upon distributing a flexible skin tactile sensor array on the ‘Baxter’ robotic forearm, the real-time human touching detection accuracy reached 96% [18]. Moreover, by utilizing a piezoelectric force sensor matrix, the gesture recognition accuracy of a ‘smart glove’ could reach ~98%. In addition to the tactile sensor matrix, Inertial Measurement Units (IMU) are also renowned in wearable devices due to their compact size, high resolution, fast response, low cost, and compatibility with different systems [19]. The synergy of multiple IMUs led to successful applications in gesture recognition [20], dance mimics [21], gait analysis [22], tumble detection [23], daily life activity classifications [24], etc.

Intention recognition is another aspect of HMI, which refers to the prediction of human activities based on sensor output data [25]. In practice, the intention-recognition accuracy is affected by factors such as the resolution of the sensor, the install location of the sensor, the complexity of the gesture/action, and the types of sensors synergized for prediction [19]. In addition to sensor selection and setup, a data processing and intention prediction model is also crucial for intention-recognition accuracy. In recent years, the research on intention prediction has mainly focused on the following approaches: Statistics [26,27], machine learning [10,28], and deep learning [29,30,31,32]. Representative statistic approaches such as the least-squares method and the Kalman filtering algorithm possess advantages such as low computational complexity and good real-time control performance. However, to achieve high prediction accuracy, a linear correlation is required between data captured by the sensor and the demanded action trajectory [33]. In other words, the statistical approach is only applicable to simple motion prediction. To address this issue, machine learning and deep learning methods have been extensively studied. Representative machine learning approaches such as the Maximum Entropy Markov Model (MEMM) and the Support Vector Machine (SVM) usually require heavy data pre-processing such as Wavelet Transform (WT) or Principal Component Analysis (PCA) to optimize eigenvalues of the data sets [10,28]. Although the popular SVM model can make reasonable predictions on data sets with non-linear correlations, compared to deep learning methods such as the Convolutional Neural Network (CNN), more computational time is usually required for large sample sizes [34], and the prediction accuracy of SVM is more sample-size-dependent, due to its inferior feature-extracting capability [34].

To assist users in rehabilitation and daily life activities, a reliable mechanical structure design of hand exoskeletons is indispensable. Based on the force transmission mechanism, the hand exoskeleton can be classified as pneumatic [35], cable/tendon-driven [36,37], smart-material-based artificial muscle-driven [38,39,40], and linkage-driven [41,42] technology. ‘Stiff hand’ is usually observed in stroke patients, and significant torque force is required to perform successful rehabilitation. Artificial muscles based on smart materials such as dielectric elastomers [39] and electroactive polymers [40] are not applicable as they are usually insufficient in the generation of power, force, and deformation. Due to the compressible and temperature-sensitive nature of gas, the bending angle and bending speed of each finger joint cannot be precisely controlled by a pneumatic ‘muscle’ [43]. The cable-driven design reduces the weight of the exoskeleton. In practice, the cables and artificial tendons usually experience elastic deformation in operation, which may require constant calibration to avoid misalignment with the rotation center of finger joints [44,45]. Most existing cable-driven designs only drive the fingers through the stretch or bend phase, and the complete bend–stretch process cannot be repeated without the intervention of additional complex mechanisms [46]. Overall, soft design, which involves pneumatic ‘muscle’, artificial tendon, or smart material, provides a comfortable wear experience; however, most exoskeletons with a low-rigidity design are heavily underactuated and one active Degree of Freedom (DoF) is usually considered for each digit, which limits its applications [47]. Compared with soft exoskeletons, this design involves linkages that are bulky and rigid, which potentially provides an uncomfortable wear experience and a heavy burden for the user [48,49,50]. Furthermore, misalignment of the finger joint (axis) and exoskeleton joint (axis) is commonly found in current designs, which potentially leads to discomfort and skin abrasion [36,51,52]. However, the linkage-driven mechanism is still widely adopted in hand exoskeletons due to the large force transmission efficiency, precise joint trajectory control potential, and reliability of the mechanism [53]. 

To facilitate post-stroke rehabilitation and provide assistance for complex daily life activities, a complete smart hand exoskeleton rehabilitation system, which covers accurate digit joints’ motion control, adjustable dimensions, a reliable intention-detection approach, and high intention-recognition accuracy, is proposed in this study. Based on the physiological structure of a human hand, a compact linkage-driven design with 10 active DoFs and 3 passive DoFs is proposed, which enables accurate control of a wide range of postures. Adopting the dimension-adjustable design, the device can be equipped by the majority of the population in the world. Based on the preferences of the user, the hand exoskeleton can be mounted on the existing up-limb exoskeleton system via a link module, which greatly diminishes the weight burden for the user. Three rehabilitation/daily life assistance modes are developed for various personal needs, namely, robot-in-charge, therapist-in-charge, and patient-in-charge modes. Considering HMI, a thin-film force sensor matrix and IMUs are installed in the exoskeleton, and the corresponding controller aims to capture/detect user intentions by tracing the force on the exoskeleton and the rotation angle of finger joints. The reliability of the sensor composition synergized with this device is assessed by the trigger rate, Kernel Density Estimation (KDE), and a confusion matrix. To recognize user intention, a genetic algorithm (GA) is applied to search for the optimal hyperparameters of CNN aiming for high intention-recognition accuracy.

## 2. Design of Hand Exoskeleton

### 2.1. Hand Skeleton Model Construction

**Figure 1:**
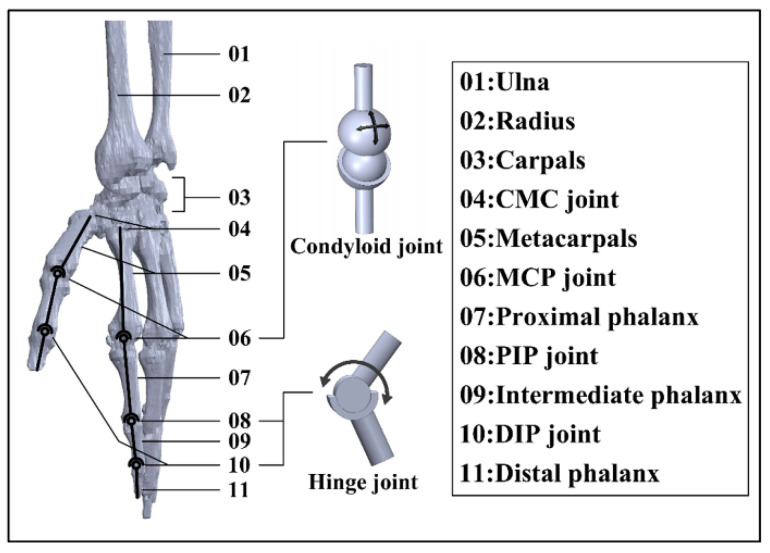
Constructed 3D model of hand skeleton based on anatomy.

The physiological structure of the hand can be revealed by analyzing the existing model of the hand skeleton in the OpenSim library. The skeleton of the hand capitates near the wrist, metacarpals, and phalanges segments. In the hand skeleton, all digits contain 1 metacarpal segment. The 4 fingers have 3 segments, namely, proximal, intermediate, and distal phalanxes. The thumb possesses 2 phalanx segments, which are proximal and distal phalanxes. The joints of the hand are named according to the bones to which they connect. Consequently, there is 1 metacarpophalangeal joint (MCP), 1 distal interphalangeal joint (DIP), and 1 proximal interphalangeal joint (PIP) for the 4 fingers, while the thumb contains only 1 MCP and 1 DIP joint (Figure 1). In addition, there is a carpometacarpal joint (CMC) for each digit near the wrist. 

The joint between each phalanx can be treated as a 1 DoF hinge joint, as 2 phalanges can only bend and extend along the vector direction shown in Figure 1. The MCP joint is equivalent to 2 DoFs, a ball-and-socket model that can rotate along the two directions. The CMC joints can be regarded as a 2 DoF saddle joint [54]. All digits in one hand have a total of 29 DoFs, where the thumb contains 5 DoFs and each of the four fingers has 6 DoFs. If all 29 DoFs are adopted as active DoFs, the weight of the hand exoskeleton device would be a huge burden and the reliability of the device in both motion transmission and motion control would be low. To carry out a successful grasp, each digit acts independently for flexion–extension, and the trajectory of each joint is constrained in a single plane. 

Notably, the four fingers and the thumb do not share the same physiological structure. The intermediate phalanx is absent for the thumb. Moreover, the DIP joint of the thumb possesses a significantly larger active rotation range (compared with the DIP joints of fingers). However, the CMC joint (especially the CMC of the thumb) plays an essential role in grasping in terms of flexibility and force transmission. The simple grasp action can be performed with all metacarpals fixed, and rotation of the CMC joint is not mandatory. Therefore, the hand exoskeleton designed in this paper only considers the DoFs required by flexion–extension, which mainly involves PIP and MCP joints for the four fingers, while DIP and MCP joints are considered for the thumb. 

### 2.2. Finger Kinematics

In order to conduct a finger kinematics analysis, the measurement of a volunteer is necessary. Measurements are conducted on phalanges and metacarpals with the aid of a vernier caliper. These measurements are recorded in Table 1. Note that while the exoskeleton is developed based on a single subject, the fitness for a larger population is considered, which is thoroughly discussed in Section 2.3.1. An experiment on subjects with different hand sizes is presented in Section 3.2.3. 

**Figure 2:**
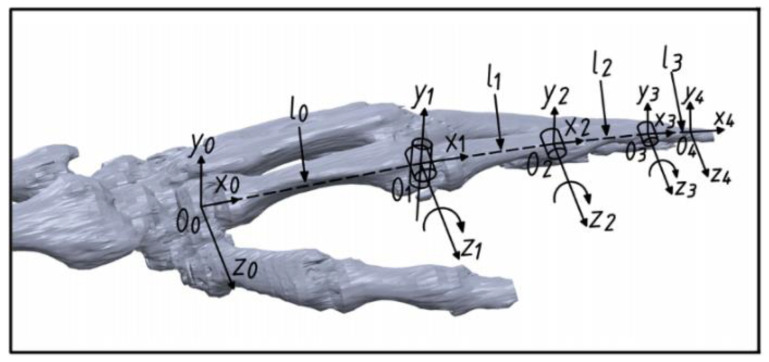
Coordination system of the 3D hand model.

Considering all the possible gestures/actions performed by the hand, the skeleton of the hand plays an essential role in posture support, and the length of each digit stays approximately the same during the rotation process. Taking the index finger as an example, we treat the metacarpal bone as a fixed base frame and the metacarpal bone, proximal, middle, and distal phalanxes form an open-chain four-linkage mechanism. For a grasping action, the 3 DoFs in the four-linkage mechanism are all rotational, and the rotation angle ranges are 0–90°, 0–110°, and 0–70° for MCP, PIP, and DIP, respectively. Based on a modified hand skeleton model (Figure 2), D-H parameters (Table 2) of the equivalent four-linkage mechanism are established to study the kinematics of the hand, where O_0_ is the coordinate system fixed at one end of the metacarpal close to carpals (CMC joint) and the O_1_, O_2_, O_3_, and O_4_ coordinate system is located at the geometric center of the MCP, PIP, DIP, and fingertip, respectively.

In this research, the hand exoskeleton is designed to carry out rehabilitation training and aid patients in daily life activities such as object grasping. To implement the grasp action, muscles and tendons drive the MCP joint first, followed by PIP and DIP joints. In this study, workspace refers to the collection of spatial positions that a joint can reach under constraints. 

**Figure 3:**
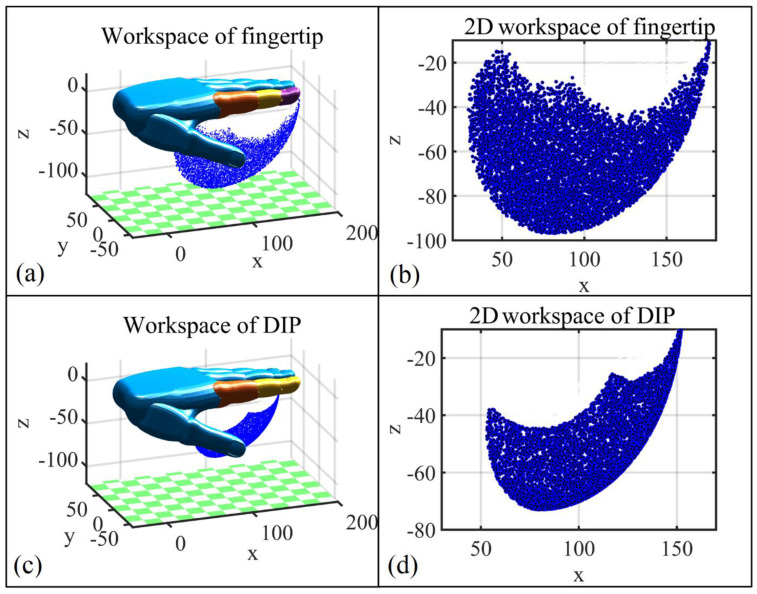
The workspace of the tip and DIP joint of the index finger. (**a**) The workspace of the index fingertip; (**b**) 2D view of the workspace of the index fingertip; (**c**) The workspace of the DIP joint; (**d**) 2D view of the workspace of the DIP joint.

Based on the Monte Carlo method [55], workspace studies on the fingertip and (the geometric center of) the DIP joint are carried out first to build and validate the initial design of the hand exoskeleton. Random valid rotation angles of each joint are substituted into a kinematics matrix based on the D-H setup to obtain the workspace cloud map of the index fingertip and DIP joint (Figure 3). As can be seen, the workspace of the DIP joint lays inside the workspace of the fingertip; however, the relatively smaller DIP joint workspace is enough for grasping large objects such as a bottle.

### 2.3. Design of the Exoskeleton Structure 

#### 2.3.1. Structure Analysis

**Figure 4:**
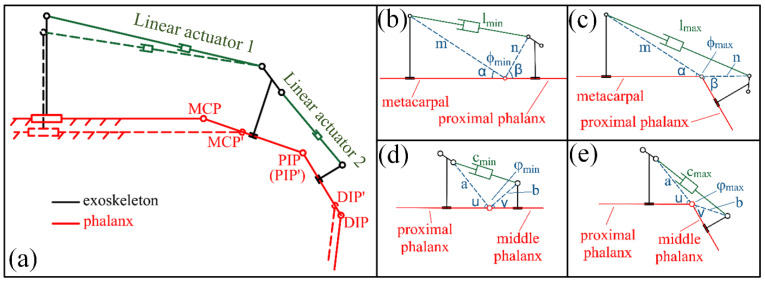
Sketch of exoskeleton structure for index finger. (**a**) Four-linkage mechanism of exoskeleton; (**b**) minimum angle of MCP joint; (**c**) maximum angle of the MCP joint; (**d**) minimum angle of PIP joint; (**e**) maximum angle of the PIP joint.

In order to perform rehabilitation exercises or grasp activities, the flexion–extension motion for each digit is essential. Among all the force transmission mechanisms for rehabilitation exoskeletons, the four-linkage mechanism is simple and accurate for motion control, and thus is adopted in this study. Figure 4a presents the schematic diagram of the exoskeleton mechanism for the index finger. Rotation mechanisms for the MCP and PIP joints in the hand exoskeleton are the same. Taking the MCP joint as an example first, the metacarpal bone serves as a fixed-base frame and the proximal phalanx functions as a phantom element; together, they form a closed-chain mechanism with linear actuator 1 and exoskeleton linkages. Linear actuator 1 is an active member and dominates the flexion–extension behavior of MCP. Four constant parameters, m, n, α, and β labeled in Figure 4b,c, are adopted to describe the relationship between the actuator and angle ϕ. The relation between the length of the linear actuator 1 (l) and ϕ can be expressed as:

(1) $$cos\phi =\frac{m^{2}+n^{2}-l^{2}}{2mn}$$

The rotation angle of the MCP joint can be presented as ψ=α+β+ϕ−π. Similarly, a, b, u, and v are adopted for PIP joint-related rotation and the rotation angle of the PIP joint ω=α+β+ϕ−π. Grasping activities in daily life does not require the full rotational range of joints. To hold a cup with a diameter of ~10 cm, the angular rotation in MCP and DIP is ~10° and ~45°, respectively, for the thumb, while the MCP and PIP joints rotate ~30° and ~60°, respectively, for the rest of the 4 fingers. In this consideration, the maximum rotation angle for MCP and PIP joints is designed to be 60°, which guarantees the safety of users and fulfills the needs for activities such as grasping and rehabilitation. With the above-mentioned understanding, Figure 4b,c illustrate the minimum and maximum lengths of the linear motor 1; meanwhile, Figure 4d,e show the minimum and maximum PIP joint rotation angles, respectively.

**Figure 5:**
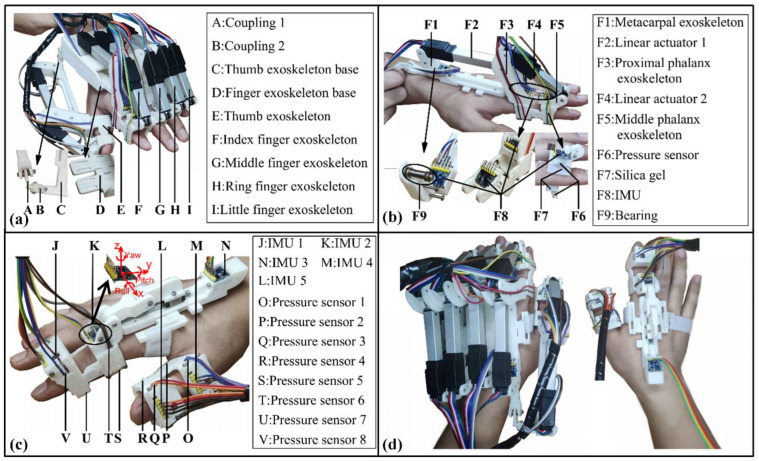
Hand exoskeleton rehabilitation system. (**a**) Overview of the hand exoskeleton; (**b**) components in the index finger exoskeleton; (**c**) detailed illustration of the wearable controller; (**d**) the synergy of hand exoskeleton and the wearable controller.

**Figure 6:**
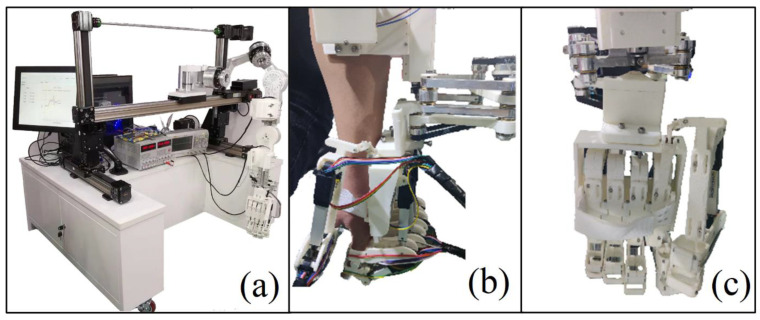
The combination of the hand exoskeleton and the existing upper-arm rehabilitation system. (**a**) Picture of the whole system; (**b**) side view of the hand exoskeleton mounted on upper-arm rehabilitation system; (**c**) bottom view of the hand exoskeleton mounted on upper-arm rehabilitation system.

The overall design of the hand exoskeleton is presented in Figure 5a. Based on the preferences of the patient and suggestions of the doctor, the hand exoskeleton can either function independently or perform rehabilitation with support from the existing upper-limb exoskeleton system presented in Figure 6. The hand exoskeleton can be attached to the arm exoskeleton via a link module, which greatly diminishes the weight of the hand exoskeleton that a user needs to bear. Most components of the hand exoskeleton are realized via 3D printing utilizing polylactic acid (PLA), which is a low-density material. The strength of essential parts is verified via the FEA method (Supplementary Material, Figure S1). The structure strength meets the requirements of tasks such as rehabilitation and low-weight object holding.

The hand exoskeleton contains 10 active DoFs and 3 passive DoFs in total. The motion of each digit can be controlled separately. Taking the index finger exoskeleton as an example (Figure 5b), there are 2 linear actuators selected for joint rotation, which are FIRGELLI L12-50-100-12-I (linear actuator 1) and L12-30-100-12-I (linear actuator 2). All 3 passive DoFs are shown in the insert of Figure 5a, aiming to adjust the relative position between the finger exoskeleton base and the thumb exoskeleton base. The rotation of passive joints can be constrained by tightening the bolts when comfortable angles are found for rehabilitation and grasping. More information regarding passive joints is presented in Supplementary Materials, Figure S2.

Consider the interaction and force transmission between the hand and wearable exoskeletons, fingers and exoskeleton are tightened by the presence of elastic silica gel (inset of Figure 5b). The exoskeleton rotation axes and finger rotation axes are lined up to minimize the possible relative sliding between exoskeleton linkage and human phalanges. Regarding the friction between linkages and actuators, miniaturized bearings are adopted. For each digit, 4 thin-film pressure sensors are sandwiched between the silica gel (both dorsal and palmar sides) and digit holder. In addition, 3 IMUs are installed in the exoskeleton in the labelled position of Figure 5b. Adopting the design of the hand exoskeleton, a wearable controller without an actuator is assembled with a pressure sensor and IMUs installed in the positions labelled in Figure 5c. This wearable controller is designed for HMI, which is thoroughly discussed in Section 3.

Although the hand exoskeleton is developed based on one subject, the fitness of people with different phalanx/digit lengths is considered in this design. Representative anthropometric data are considered first; however, a complete and convincing segment data sheet is rare in the literature. Thus, the phalanx lengths of 20 subjects were measured. The height of subjects ranged from 152 to 191 cm. Based on the measurements, a length-adjustment mechanism was designed. To ensure a comfortable rehabilitation experience for different people, the rotation axes of the PIP joints (DIP joint for the thumb) of the hand exoskeleton and the human hand need to be aligned first and then the length of the metacarpal exoskeleton is adjusted via the sliding chute of the metacarpal exoskeleton (Supplementary Materials, Figures S3–S5) to align the rotation axis of the MCP joints (Figure 4a). The metacarpal exoskeleton, linear actuator 1, and proximal phalanx exoskeleton together form an open-chain mechanism. The metacarpal exoskeleton needs to be fixed to the human hand (via either a bandage or glove) to ensure the accurate control of joints. 

**Figure 7:**
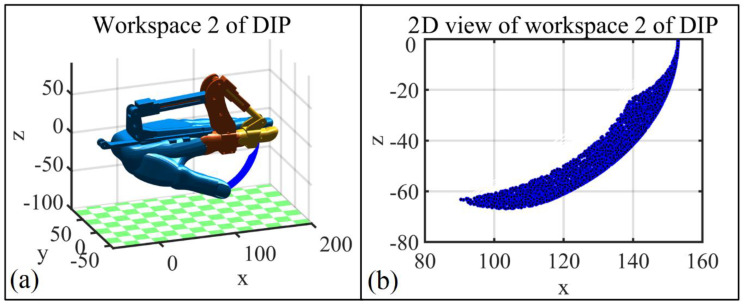
Workspace of exoskeleton worn by index finger. (**a**) The DIP joint workspace of the index finger driven by index finger exoskeleton; (**b**) 2D view of the DIP joint workspace for the corresponding index finger exoskeleton.

Based on the Monte Carlo method, Figure 7 represents the DIP joint workspace (A) of the index finger, which is driven by the exoskeleton. Considering the entire workspace B of the DIP joint (Figure 3d), the two workspaces present the following relationship A⊂B, which guarantees the safety of the exoskeleton user in all circumstances.

#### 2.3.2. Kinematic Analysis

To execute rehabilitation training or grasp tasks precisely, joint space trajectory planning is needed to describe each joint angle variation with respect to time. Moreover, angular velocity and angular acceleration of both MCP and PIP joints during the rotation process need to be constrained to avoid the possibility of finger injury. To guarantee a gentle acceleration for each finger joint, a quintic polynomial is adopted for the trajectory planning of each joint. The quintic polynomial contains 6 coefficients ($C_{0},\;C_{1},\;C_{2},\;C_{3},C_{4},\;C_{5}$), which constrain the angle, angular velocity, and angular acceleration. The corresponding angle, angular velocity, and angular acceleration of both joints meet the following requirements:

(2) $$\{\begin{array}{c} \psi \left(t\right)=C_{0}+C_{1}t+C_{2}t^{2}+C_{3}t^{3}+C_{4}t^{4}+C_{5}t^{5}\\ \psi^{\prime }\left(t\right)=C_{1}+2C_{2}t+3C_{3}t^{2}+4C_{4}t^{3}+5C_{5}t^{4}\\ \psi^{\prime \prime }\left(t\right)=2C_{2}+6C_{3}t+12C_{4}t^{2}+20C_{5}t^{3} \end{array}$$

We assume 10 s is required for MCP and PIP joints to rotate 60°, taking $t_{0}$ and $t_{e}$ as the start and end time for both joints, and the 6 parameters in Equation (3) are presented as follows:

(3) $$\{\begin{array}{c} C_{0}=0\\ C_{1}=\psi^{\prime }\left(t_{0}\right)\\ C_{2}=\frac{\psi^{\prime \prime }\left(t_{0}\right)}{2}\\ C_{3}=\frac{\psi^{\prime \prime }\left(t_{e}\right)}{20}-\frac{3\psi^{\prime \prime }\left(t_{0}\right)}{20}-\frac{3\psi^{\prime }\left(t_{0}\right)}{50}-\frac{{\psi^{\prime }}^{\left(t_{e}\right)}}{25}+\frac{\pi }{300}\\ C_{4}=\frac{3\psi^{\prime \prime }\left(t_{0}\right)}{200}-\frac{\psi^{\prime \prime }\left(t_{e}\right)}{100}+\frac{\psi^{\prime }\left(t_{0}\right)}{125}+\frac{7{\psi^{\prime }}^{\left(t_{e}\right)}}{1000}-\frac{\pi }{2000}\\ C_{5}=\frac{\psi^{\prime \prime }\left(t_{e}\right)}{2000}-\frac{\psi^{\prime \prime }\left(t_{0}\right)}{2000}-\frac{3\psi^{\prime }\left(t_{0}\right)}{10000}-\frac{3{\psi^{\prime }}^{\left(t_{e}\right)}}{10000}+\frac{\pi }{50000} \end{array}$$

**Figure 8:**
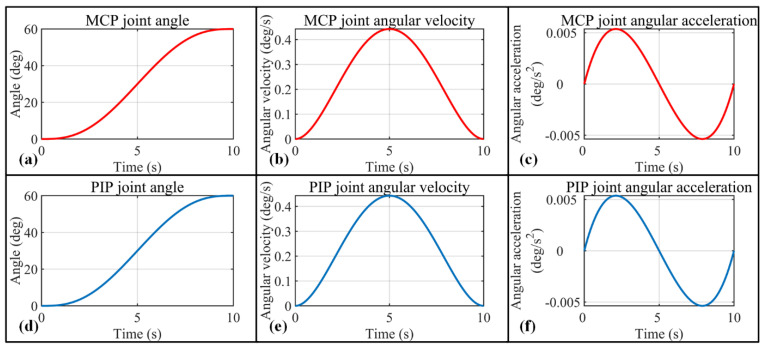
Trajectory planning for index finger exoskeleton MCP joint and PIP joint. (**a**–**c**) MCP joint angle, angular velocity, and angular acceleration variation with respect to time; (**d**–**f**) PIP joint angle, angular velocity, and angular acceleration variation with respect to time.

**Figure 9:**
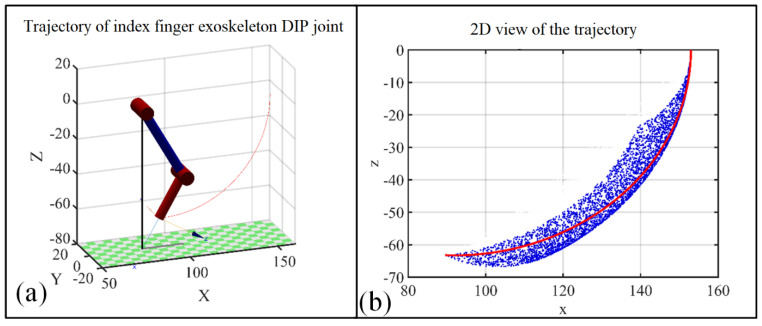
Trajectory of index finger exoskeleton DIP joint to accomplish a grasp action; (**a**) 3D view of the trajectory; (**b**) trajectory of index finger exoskeleton DIP joint compared with the workspace of the DIP joint.

To guarantee gentle and stable rehabilitation training with the exoskeleton, the speed and acceleration of the MCP and DIP joints are set to 0 for the start and end points. Based on the setup above, angle, angular velocity, and angular acceleration changes with respect to time are calculated for MCP and PIP joints, which are presented in Figure 8. Figure 9a presents the trajectory of the corresponding (index finger exoskeleton) DIP joint, and as can be seen, the trajectory exists completely inside the workspace of the index finger exoskeleton DIP joint (Figure 9b).

To ensure the fingers under the control of the exoskeleton move according to the previously determined trajectory, it is necessary to control the linear actuator precisely. Based on Figure 4 and Equation (1), the length of linear actuators 1 and 2 (Figure 5b) can be expressed as:

(4) $$\{\begin{array}{c} l\left(t\right)=\sqrt{m^{2}+n^{2}-2mn\;cos\left[\pi -\alpha -\beta +\psi \left(t\right)\right]}\\ c\left(t\right)=\sqrt{a^{2}+b^{2}-2ab\;cos\left[\pi -u-v+\psi \left(t\right)\right]} \end{array}$$

**Figure 10:**
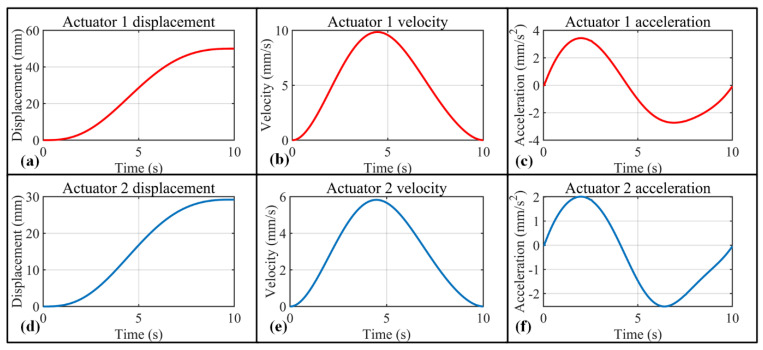
Displacement, velocity, and acceleration diagrams of the two actuators in order to rotate 60° in 10 s for MCP and PIP joints. (**a**) Displacement–time diagram of actuator 1; (**b**) velocity–time diagram of actuator 1; (**c**) acceleration–time diagram of actuator 1; (**d**) displacement–time diagram of actuator 2; (**e**) velocity–time diagram of actuator 2; (**f**) acceleration–time diagram of actuators 2.

For the grasp action defined in this section, the displacement, velocity, and acceleration for linear actuators 1 and 2 are presented in Figure 10.

## 3. Hand Exoskeleton HMI Strategies

### 3.1. Hand Exoskeleton System Overview

**Figure 11:**
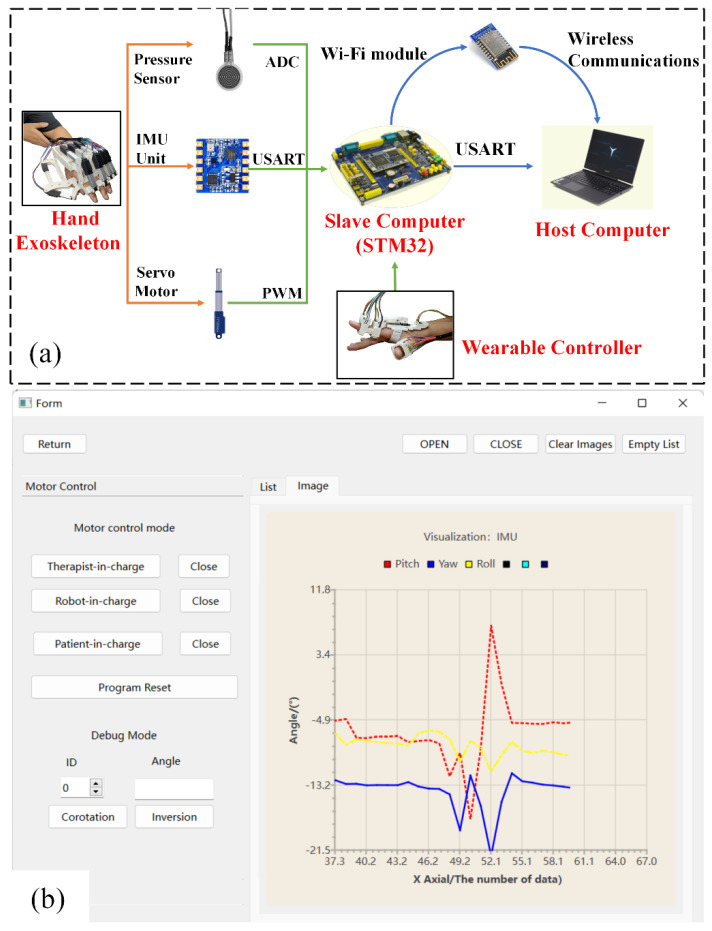
Control of the hand exoskeleton rehabilitation. (**a**) System overview of the hand exoskeleton; (**b**) interface program to control the hand exoskeleton.

The overall control system is composed of four major parts, including the hand exoskeleton, host computer, slave computer (STM32-F329 microcontroller, manufactured by Zhengdianyuanzi Ltd., Guangzhou, China), and a wearable controller (Figure 11a). The host computer processes data collected by the slave computer and sends commands via the interface program developed in the QT environment. As shown in Figure 11b, the interface program possesses two basic functions including mode selection and data visualization. The slave computer integrates one analog-to-digital converter (ADC) and one serial port transmission module and controls the linear actuator via pulse width modulation (PWM). 

Considering the high real-time and high-resolution requirements for rehabilitation, thin pressure sensors (RP-C18.3-ST, manufactured by Aodong Ltd., Dunhua, China) and IMUs (IMU901, manufactured by Zhengdianyuanzi Ltd.) are selected for human–machine interaction, and the distribution of these sensors is illustrated in Figure 5. The thin-film pressure sensors selected are piezoelectric and their pressure reading can be calibrated via the resistance–voltage conversion relation:

(5) $$U_{0}=\left(1+R_{AO-RES}\times \frac{1}{R_{x}}\right)\times 0.1$$

where $R_{AO-RES}$ represents the adjustable resistance and $R_{x}$ is the resistance that changes in real time with respect to pressure changes. The real-time pressure data collected by the sensor can be converted into an analog voltage (0~3.3 V) through the ADC module in the slave computer. The adopted IMU integrates a gyroscope, accelerometer, magnetometer, and barometer. The IMU outputs the variation of pitch, roll, and yaw angles via the Universal Synchronous Asynchronous Receiver Transmitter module (USART). In order to minimize the interference of ‘abnormal data’ (induced by shaking of the hand, random motion of the arm, etc.) while ensuring the reliability of data, an amplitude-limiting filtering algorithm (integrated into STM32) is utilized to constrain the steep variation in the data.

### 3.2. Control Modes for Rehabilitation and Daily Life Activity Assistance

Stroke patients usually need a long rehabilitation period after surgery in order to recover from stroke-related complications such as hemiplegia. Patients’ demands at different rehabilitation stages vary even for the same patient [14], thus, rehabilitation therapy should also be changed accordingly. Regarding this issue, human–machine interaction (HMI) technology is adopted to adjust rehabilitation therapy and control the motion of the hand exoskeleton based on personal needs. Three modes are designed for rehabilitation and daily life assistance, namely, robot-in-charge, therapist-in-charge, and patient-in-charge modes. 

The robot-in-charge training strategy aims to help patients without the ability to move or exercise. In this mode, the hand exoskeleton guides the patient’s hand along a pre-planned path (proposed by doctors). The therapist-in-charge training strategy is suitable for patients in all recovery stages and requires a therapist to put on the wearable controller (Figure 5c). The angular rotation of the therapist’s hand is mapped onto the patient’s hand via tracking pitch, roll, and yaw angles obtained by IMUs. The patient-in-charge training strategy targets patients who are capable of low-intensity exercises. In this mode, two functions can be achieved, which are rehabilitation and daily activity assistance. A wearable controller is required to be worn by one hand, while the hand exoskeleton is equipped with the other hand (Figure 5d). Utilizing deep learning and machine learning methods, data (collected by the wearable controller) can be correlated to different pre-planned exoskeleton postures/actions. More information regarding the three modes is presented in the following sections.

#### 3.2.1. Robot-in-Charge Rehabilitation Mode

**Figure 12:**
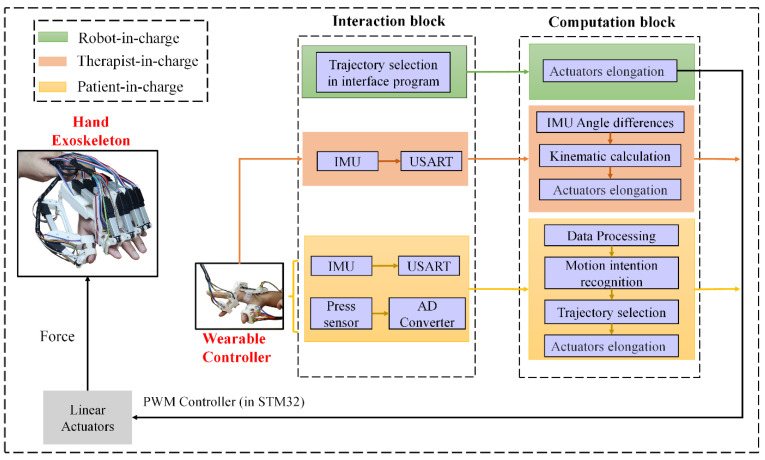
Hand rehabilitation exoskeleton control flow diagram for the three rehabilitation modes.

Figure 12 illustrates the control flow diagram for the three rehabilitation modes, where the robot-in-charge mode is presented by green blocks. Based on the rehabilitation therapy suggested by the doctor, the trajectory of each exoskeleton joint can be planned with the aid of Equation (2), and the corresponding elongation in the linear attractors is calculated via Equation (4). In the rehabilitation process, the real-time data collected by the thin-film pressure sensors installed in the hand exoskeleton (Figure 5b) can be monitored by doctors and the data can be used as a recovery evaluation index.

#### 3.2.2. Therapist-in-Charge Rehabilitation Mode

The therapist-in-charge training mode is presented by the orange blocks in Figure 12. In this mode, a wearable controller is required to be equipped by the therapist. In Figure 5c, there are three IMUs (IMUs 1–3) that record the rotation of the index finder, while two IMUs (IMU 4–5) are installed to detect the motion of the thumb. The three angle readings (pitch, roll, and yaw, specified in Figure 5c) from IMU 3 mainly serve the purpose of a motion benchmark, and the rest of the angles function for hand exoskeleton motion tracking. 

In the rehabilitation process, rotation in the index finger PIP or MCP joint leads to the angle variation in IMUs 1 or 2, respectively, while motion in the thumb MCP or DIP can be detected by IMUs 4 or 5, respectively. IMUs in one digit are all aligned in the same plane. For any adjacent two IMUs, differences in pitch and yaw angles are expected to be 0. Taking the index finger PIP joint as an example, using the reading of IMU 2 as a benchmark, the PIP rotation angle can be expressed as follows:

(6) $${\left[\begin{array}{c} Roll\\ Pitch\\ Yaw \end{array}\right]}_{MCP}={\left[\begin{array}{c} Roll\\ Pitch\\ Yaw \end{array}\right]}_{Imu1}-{\left[\begin{array}{c} Roll\\ Pitch\\ Yaw \end{array}\right]}_{Imu2}$$

**Figure 13:**
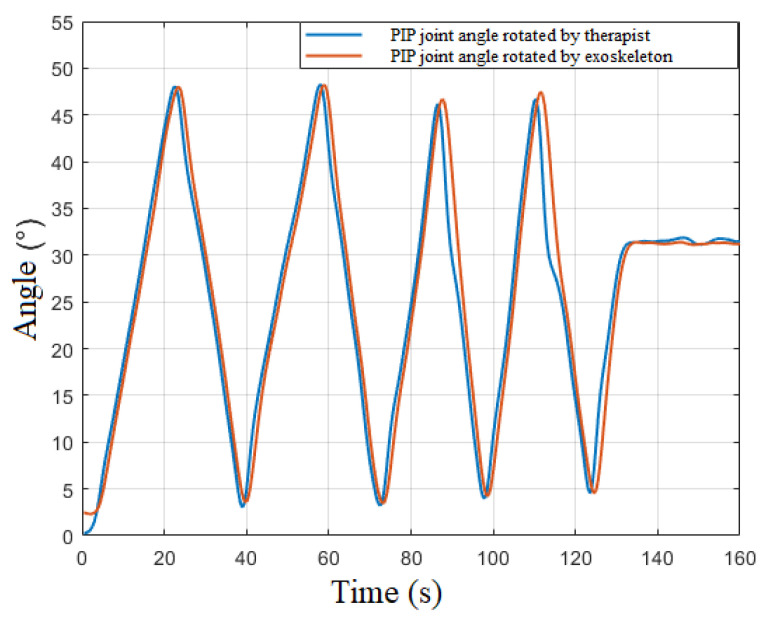
Real-time PIP joint angles variation for index finger of therapist and index finger exoskeleton. Blue line refers to the PIP joint rotation performed by therapist equipped with wearable controller, while the red line presents the PIP joint angle change in index finger exoskeleton.

With the aid of the slave computer, the real-time PIP joint angle variation of the therapist’s index finger is obtained. IMUs installed in positions of the hand exoskeleton are similar to the positions in the wearable controller (Figure 5b), and the angle variation in each joint of the hand exoskeleton can also be calculated with the aid of Equation (5). Utilizing Equation (4), the demanded elongation of the linear actuator installed in the exoskeleton is calculated. Figure 13 indicates the decent real-time performance of the therapist-in-charge training mode.

#### 3.2.3. Patient-in-Charge Rehabilitation Mode 

The patient-in-charge training strategy designed in this research targets patients with limited exercise ability who are only able rotate digit joints at a small angle (e.g., 5°). For these patients who require self-rehabilitation and complex daily activities, intention recognition is of vital importance. ‘Stiff hand’ is usually observed in stroke patients, and the stiffness is unpredictable considering the vast population of stroke patients, thus recognizing one hand’s posture/action to guide the other hand’s motion is the best strategy. In this study, both the exoskeleton and its corresponding controller are adopted.

**Figure 14:**
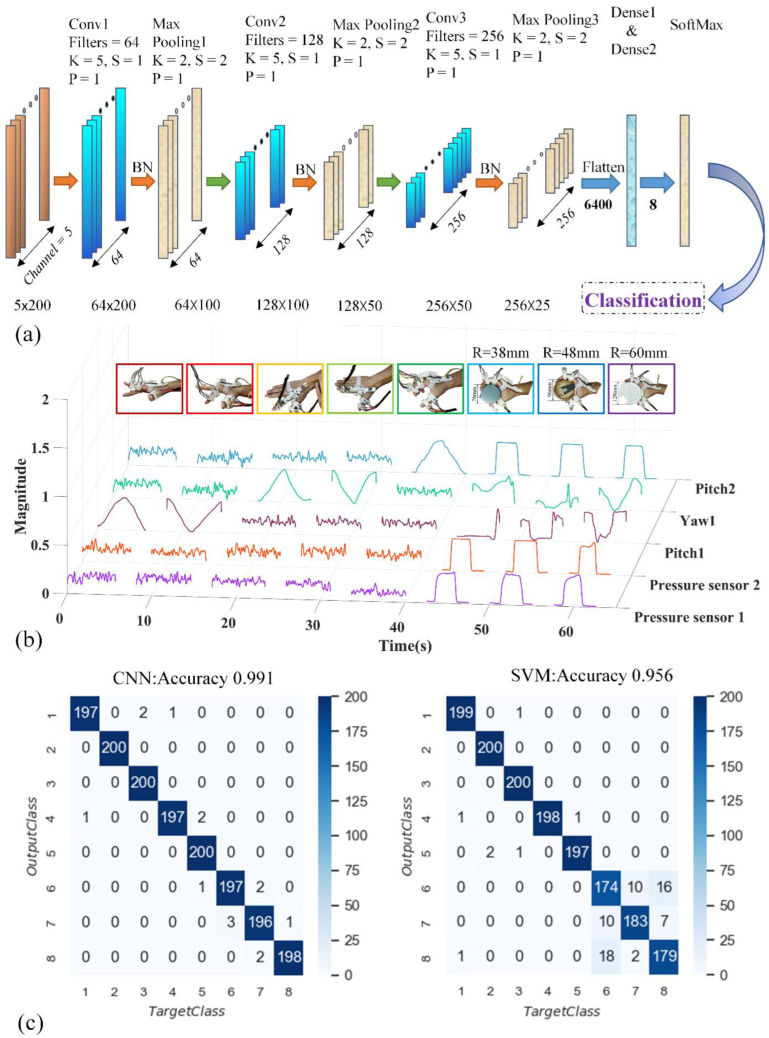
Deep Learning and machine-learning-based intention recognition. (**a**) 1D CNN structure diagram; (**b**) sensor output data pattern of the six actions/gestures; (**c**) confusion matrix diagrams of CNN (**left** panel) and SVM (**right** panel) models.

Compared with statistical intention-recognition methods, the deep learning approach of a CNN (Figure 14a) is adopted for its renowned training efficiency and prediction accuracy [19]. Results of the CNN model are validated and compared with the widely adopted machine learning method SVM. Gestures of the hand recognized by the wearable controller can be correlated with planned trajectories of the hand exoskeleton, and these trajectories can be planned and adjusted based on the needs of patients, utilizing Equations (2)–(4).

##### Data Acquisition and Processing

In this research, the wearable controller is worn by the right hand of volunteers to record data from both the IMUs and thin-film pressure sensors labelled in Figure 5c. Eight unique gestures/actions are selected for the identification experiment (inset of Figure 14b). Five healthy volunteers are involved in the data acquisition (the hand size of each volunteer is presented in Supplementary Materials Figure S5), and each gesture/action is repeated 250 times by each individual volunteer. A total of 10,000 sets of data are collected. Among all the data sets, a random 84% are utilized for training and the remaining 16% are used for testing. To mimic a real application scenario and improve intention-recognition accuracy, diversity of data sets for each gesture/action is necessary. In other words, even for the same gesture/action, the rotation angle (0 to ~60°) for each joint and the force (0 to ~3 N) exerted on the pressure sensor varies significantly for each individual repeat. In addition, during the data-acquisition process, random movement of the arm is inevitable. As such, IMU also records information related to arm rotation, shaking hand, etc. Prior to data acquisition, IMUs and pressure sensors are calibrated. The data acquisition frequency is fixed at a low value of 40 Hz, and each individual gesture/action is performed at a slow pace, which guarantees the diversity of data sets. For the actual rehabilitation process, the data collection frequency can be adjusted based on the preferences of the user.

In the data acquisition phase, the first two gestures are performed by rotating the z1 axis (Figure 2) in counterclockwise and clockwise directions, respectively. The third and fourth gestures/actions are achieved by rotating the y1 axis (Figure 2) in clockwise and counterclockwise directions, respectively. The fifth gesture refers to the bending of both MCP and PIP joints in the index finger. The last three gestures/actions are holding cylinders with a small radius (38 mm, 48 mm, and 60 mm, respectively), aiming to test the effectiveness of the whole HMI strategy. Each collected data set contains five columns, with 200 data points fitted in each column. The first two columns record measurements from pressure sensors 5 and 7 labelled in Figure 5c. The third column refers to the pitch angle change in IMU 2, which describes the up- and down-motion of the index finger dominated by the MCP joint. The roll angle variation of IMU 2 is recorded in column 4, aiming to distinguish between left and right MCP rotation. For the fifth column, the pitch angle difference between IMU 1 and IMU 2 is taken for the description of PIP joint rotation. 

Data processing techniques such as normalization and feature extraction are essential for deep learning and machine learning models. Considering the range of measurements from distinct sensor types and the distribution patterns of each data set [56], extra efforts may be required for the CNN model to balance the multiple distribution centers if normalization is not applied. As a result, it slows down the training efficiency, also making the model more difficult to converge. Normalization is achieved in two steps. Firstly, all numbers in each column are scaled to fit in the range of [0, 1], utilizing $\frac{\left(x-x_{Min}\right)}{x_{Max}-x_{Min}}$. Then, the data set mean value is adjusted to 0 based on $\frac{\left(x-\mu \right)}{\sigma }$. In addition, effective feature extraction reduces the correlation of irrelevant dimensions in the data sets, thereby speeding up the training process [57]. Regarding the feature extraction process, it can be achieved in the convolutional layer of the CNN model. For the SVM model, Principal Component Analysis (PCA) is required to reduce the dimensions of the original data set.

##### Intention-Recognition Model and Results

The structure of the one-dimensional CNN deep neural network model adopted in this research is shown in Figure 14a, which is mainly composed of convolutional, batch normalization, pooling, SoftMax, and fully connected layers. The convolutional layer is designated to extract the features of the specified data segment. The batch normalization layer ensures a decent backpropagation gradient, which alleviates the problem of vanishing gradients [58]. The pooling layer is presented for the reduction of input matrix dimensions. The SoftMax layer stabilizes the values in the backpropagation process and leads to easier convergence for the classification task. The fully connected layer links all the previous features to obtain the classification result. The key parameters, Filters (F), Kernel size (K), Strides (S), and Padding (P), are presented in Supplementary Materials Table S1. In addition to the parameters mentioned above, the training result of the CNN model is also sensitive to the variation of hyperparameters. In the consideration of intention-recognition accuracy, GA is adopted to find the optimal hyperparameters. GA is a set of mathematical models abstracted from the process of reproduction in nature. It realizes the heuristic search of complex space by simplifying the genetic process. The flow chart of GA (more specifically, the differential evolution algorithm) is shown in Figure S6 (Supplementary Materials). The average recognition accuracy of 10-times K-fold cross-validation is taken as the fitness function of individuals in the population, and the three hyperparameters (Learning Rate, Batch Size, and Epoch) are taken as the decision variables in Table S2 (Supplementary Materials). After 10 generations of population iterations, the optimal parameters of the model were obtained and are shown in Figure S7 and Table S3 (Supplementary Materials).

SVM is a widely adopted machine learning method for classification and intention recognition. The performance of the SVM model is highly related o three hyperparameters, which are kernel function, penalty parameter C, and Gamma. In this study, the linear data dimension reduction algorithm PCA retains 98% of the key information in the original data sets, which minimizes the information loss while compressing data set dimensions significantly and accelerating training/testing. PCA processing reduces each sample data set’s dimensions from 1 × 1000 to 1 × 27. The genetic algorithm is also utilized to optimize the hyperparameters of the SVM model. The average recognition accuracy of 10-times K-fold cross-validation is also taken as the fitness function of population individuals. The three hyperparameter parameters mentioned above (kernel function, parameter C, and gamma) are used as decision variables in Table S4 (Supplementary Materials). After 10 generations of population iteration, the optimal parameters of the model are obtained and shown in Figure S8 and Table S5 (Supplementary Materials).

Upon adopting the optimal hyperparameters, a confusion matrix is obtained via testing data set prediction. The confusion matrix in Figure 14c indicates that both methods reach at least 95.6% overall recognition accuracy. Featuring the confusion matrix of the CNN model, each individual posture reaches at least ~98.5% prediction accuracy, and only 15 misclassifications are observed among the total 1600 testing data sets. The SVM model presents high classification accuracy for the first five postures/actions, while significant misclassifications occur when dealing with the last three cylinder-holding tasks.

## 4. Discussion

### 4.1. Mechanical Design of the Exoskeleton

**Figure 15:**
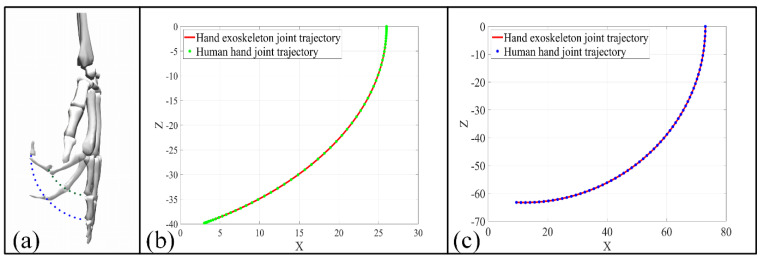
Exoskeleton trajectory validation for DIP and PIP joints. (**a**) Opensim simulation setup; (**b**) trajectory of DIP joints; (**c**) trajectory of PIP joints.

To realize accurate digit joints’ motion control mechanically, joints’ rotation axes of both the hand exoskeleton and the human hand need to be aligned in motion. To validate the concept, the trajectory of joints’ rotation axes for both the hand exoskeleton and the human hand are simulated and compared. In a scenario in which all finger joints rotate 60°, the human hand DIP and PIP joints’ trajectories obtained from Opensim fit well with the trajectories of the hand exoskeleton (Figure 15), suggesting a comfortable wear experience and potential for accurate digit joint motion control.

### 4.2. Intention Detection 

**Figure 16:**
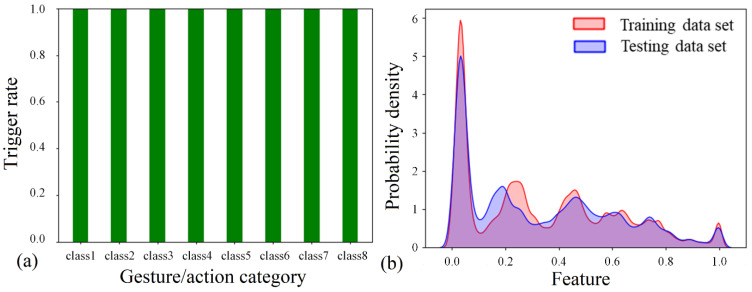
Reliability of the sensor-device synergy. (**a**) Trigger rate of all sensors necessary for gestures/actions; (**b**) probability density distribution for random training and testing data sets using KDE.

The reliability of the sensor–device synergy is assessed by the trigger rate, Kernel Density Estimation (KDE), and the confusion matrix. The trigger rate is defined by assessing the data in each data set. For a data set correlated with action 6 (grasp a cylinder with a radius of 38 mm), all five columns of data need to be considered. If all pressure sensor readings exceed 0.2 N and all angle variations exceed 2°, a successful trigger is concluded. Assessing all 10,000 data sets, a 100% trigger rate is observed for each gesture/action (Figure 16a). Moreover, good training and estimation are more likely to be achieved based on similar testing and training data set patterns. Therefore, the KDE method is applied to illustrate the probability density distribution of a random training and testing data set. As can be seen, the two distribution patterns agree well with each other (Figure 16b). The difference in distribution patterns is mainly due to the desired diversity of data sets (i.e., random motion of the arm, shaking of hands, different forces applied to the pressure sensor, different finger-bending angles, and digit length of volunteers). In addition, the high prediction accuracy for both CNN and SVM also suggests a reliable sensor–machine synergy and a good data acquisition process.

In the data-acquisition phase, each repeat is performed slowly, and 200 data points are collected, utilizing a low data collection frequency of 40 Hz. The data collection frequency of the system can be adjusted to a much higher level, which improves the overall system response time significantly. The low data collection frequency adopted in this study aims to guarantee data set diversity for training purposes. After thousands of repeats, the joint rotation in fingers cannot be controlled precisely with a high data collection frequency (due to the fatigue of the human hand), which may jeopardize the diversity of the data set. In a real rehabilitation scenario, the user may perform actions at a different pace; however, more training data sets with high diversity may help with intention-recognition accuracy. 

### 4.3. Intention Recognition

Based on the results of the confusion matrix, CNN possesses 3.5% better overall prediction accuracy compared with SVM. CNN also outperforms SVM significantly in gestures/actions 6, 7, and 8, suggesting better performance in dealing with gestures/actions with high similarities. Dividing all data sets into one training set and one testing set using the leave-one-out method may have led to biased prediction results. To validate the results, k-fold cross-validation is adopted. Selecting 10 distinct random training data sets, the network is retrained 10 times and the corresponding results are recorded in Table 3. The average accuracy of CNN presents even higher superiority over SVM with a much smaller variance presented. Three possible reasons are proposed for this phenomenon. First, CNN possesses advantages in dealing with nonlinear problems [59]. In this study, volunteers with distinct digit lengths, initial hand positions, and joint motion trajectories may lead to significant non-linear correlations between data sets and the target posture, which decreases the prediction accuracy of SVM. Secondly, the convolutional layer in the CNN model extracts more deep-level features [10], while the SVM model only extracts specific features in the data pre-processing stage (using PCA). Ideally, the first five actions/gestures all experience substantial data variation in a single column with non-periodic fluctuations observed in other columns, which are mainly due to the instability of the human hand joint, random motion of the arm, and environmental noise. As these ‘unwanted’ amplitude fluctuations exceed a certain threshold, the rate of misclassifications rises for SVM as it is unable to effectively extract the data set feature in such a scenario. Lastly, compared with the SVM model there are more adjustable parameters in the CNN model (Supplementary Materials, Tables S1 and S2), which helps it to better adapt to the eight actions/gestures in this study. Observing each individual result, the worst prediction accuracy from SVM is only 41.1% compared with CNN’s 92.2%. The low accuracy may either be a result of overfitting or the presence of substantial outliers. However, low accuracy is only observed in a single run, and outliers due to the shaking of hands and random movement of the arm are likely to be the dominant issue. Although the outliers due to arm rotation, hand shaking, etc., may violate the performance of the hand exoskeleton system, the average intention-recognition accuracy (97.1%) based on K-fold cross-validation suggests a reasonable model setup and training process. The CNN model, with its decent balance between high intention-recognition accuracy and a lightweight network structure (the prediction time consumption for both CNN and SVM models is shown in Supplementary Material Table S6), is recommended for real-time intention recognition. 

In this study, a complete hand exoskeleton rehabilitation system is proposed for post-stroke rehabilitation and assistance in complex daily life activities. Three rehabilitation/daily life assistance modes are developed for various personal needs, namely, robot-in-charge, therapist-in-charge, and patient-in-charge modes. With the aid of a sensor matrix, the patient-in-charge mode allows the detection of a small rotation angle in digits and achieves high intention-recognition accuracy when dealing with similar gestures/actions. Thus, stroke patients with limited exercise ability (e.g., 5° in each joint) can conduct self-rehabilitation and complex daily activities with the proposed device. Regarding the ‘stiff hand’ phenomenon observed in stroke patients, the synergy of the actuator (with push force up to 43 N) and linkage can provide enough torque and an accurate trajectory for digit joints. 

Note that all experiments are conducted on healthy volunteers. In future studies, the effectiveness of the hand exoskeleton system on stroke patients will be evaluated. Constrained by the size of the current electric actuator, the motion of the DIP joint is not considered. To achieve higher flexibility in the hand exoskeleton, a smaller force transmission mechanism such as voltage-sensitive composite material will be considered for the active control of finger DIP joints. The thumb CMC joint plays an essential role in grasping in terms of flexibility and force transmission. Though the current design allows the grasping of large objects (Figure S10), a mechanism with higher active DoFs for the thumb CMC joint will be designed to better service the assistive purposes. To achieve higher intention-recognition accuracy, three aspects can be considered in further study. Firstly, researchers should increase the user motion information by using more sensors in the system. Secondly, the CNN model architecture can be improved so that the model possesses stronger feature extraction capability. Thirdly, increased diversity and the number of training data sets may further improve the intention-recognition accuracy. 

## Figures and Tables

**Table 1:** Parameter for subjects’ fingers (units: mm).

	Thumb	Index Finger	Middle Finger	Ring Finger	Little Finger
**Proximal phalanx**	36	46	47	46	39
**Middle phalanx**	—	27	28	27	24
**Distal phalanx**	31	24	25	24	22
**metacarpal**	43	63	61	55	51

**Table 2:** D-H parameters for the hand skeleton in Figure 2 (units: mm).

	$l_{i}$	$\alpha_{i}$	$d_{i}$	$\theta_{i}$
***i =* 0**	80	0	0	$-\theta_{CMC}$
***i =* 1 **	46	0	0	$-\theta_{MCP}$
***i =* 2 **	27	0	0	$-\theta_{PIP}$
***i =* 3 **	24	0	0	$-\theta_{PID}$

**Table 3:** K-fold cross-validation of CNN model and SVM model.

CNN	Run number	1	2	3	4	5	6	7	8	9	10
	Accuracy	1.0	0.991	1.0	0.922	0.951	0.958	1.0	0.951	0.973	0.964
	Average	97.1									
	Variance	0.0276									
SVM	Run number	1	2	3	4	5	6	7	8	9	10
	Accuracy	0.931	0.956	0.981	0.961	0.882	0.411	0.979	0.949	0.921	0.871
	Average	0.884									
	Variance	0.162									

## Data Availability

The data presented in this study are available in this article.

## Supplementary Materials

The following supporting information can be downloaded at: https://www.mdpi.com/article/10.3390/bioengineering9110682/s1, Figure S1. Stress evaluation for thumb exoskeleton and index finger exoskeleton. Index finger exoskeleton base serves as fix frame with force (5 N each) applied in the direction labelled in red. The strength of the whole structure is also tested in real action. (a) For exoskeleton made by Aluminum 6061, the maximum stress is ~33.9 MPA compared with the material’s yielding stress; (b) for exoskeleton made by PLA material, the maximum stress is ~31.5 MPA compared with the material’s yielding stress. Figure S2. Schematic view of the three passive DoFs. Figure S3. Components in the index finger exoskeleton. There are two highlighted areas in the finger exoskeleton, which illustrate the sensor locations and the sliding chute for length adjustment. Figure S4. Hand exoskeleton worn by fingers of different phalanx lengths. (a,b) Length of proximal and intermediate phalanxes are 40 mm and 25 mm, respectively; (c,d) length of proximal and intermediate phalanxes are 46 mm and 27 mm, respectively; (e–f) length of proximal and intermediate phalanxes are 50 mm and 30 mm, respectively. Table S1. The parameters for constructing a Convolution Neural Network (CNN). Figure S5. Index finger length of the five volunteers. (a) Proximal phalanx length, middle phalanx length, and height of the volunteer are ~48 mm, ~30 mm, and ~183 cm, respectively; (b) proximal phalanx length, middle phalanx length, and height of the volunteer are ~46 mm, ~27 mm, and ~168 cm, respectively; (c) proximal phalanx length, middle phalanx length, and height of the volunteer are ~44 mm, ~24 mm, and ~170 cm, respectively; (d) proximal phalanx length, middle phalanx length, and height of the volunteer are ~43 mm, ~23 mm, and ~175 cm, respectively; (e) proximal phalanx length, middle phalanx length, and height of the volunteer are ~40 mm, ~21 mm, and ~156 cm, respectively. Figure S6. Flow chart of differential evolution algorithm. Table S2. Genetic Algorithm setup for CNN model optimization. Figure S7. Results of genetic algorithm to optimize hyperparameters of CNN model. Table S3. The optimal value of hyperparameters in the CNN model. Table S4. Genetic Algorithm setup for SVM model optimization. Figure S8. Results of Genetic Algorithm to optimize hyperparameters of SVM model. Table S5. The optimal value of hyperparameters in the SVM model. Figure S9. A demonstration of grasping objects with the passive joint setup illustrated in Figure S2. (a) A small toolbox with dimeter of ~3.5 cm; (b) water bottle with dimeter of ~6 cm; (c) Orange with dimeter of ~6 cm; A 1:35 M1A1 tank model. Figure S10. Curve of learning rate with epoch. Table S6. Prediction time using CNN model and SVM models. Figure S11. Comparison of identifiable signals with different levels of noise.
