# Supplementary material for: Hand Exoskeleton Design and Human–Machine Interaction Strategies for Rehabilitation
Source: Bioengineering (Basel). 2022 Nov 11;9(11):682. doi: 10.3390/bioengineering9110682 (PMC9687420; doi:10.3390/bioengineering9110682)
Supplement: Supplementary file 1 [file bioengineering-09-00682-s001.zip › bioengineering-2014604-supplementary.pdf]

Supplementary Materials for ‘Hand Exoskeleton Design and Human-machine Interaction Strategies for Rehabilitation’

Kang Xia [1,2]*, Xianglei Chen [1]*, Xuedong Chang [1], Chongshuai Liu [1], Liwei Guo [3], Xiaobin Xu [1], Fangrui Lv [1], Yimin Wang [3], Han Sun [3], Jianfang Zhou [1]

[1] College of Mechanical & Electrical Engineering, HoHai University, Nanjing 210098, China
[2] School of Mechanical, Medical and Process Engineering, Queensland University of Technology (QUT), Brisbane QLD 4001, Australia
[3] Articular Orthopaedics, The Third Affiliated Hospital of Soochow University, Changzhou, Jiangsu 213003, China
* Correspondence: xiak@hhu.edu.cn (K.X.); changei@hhu.edu.cn (X.C.)

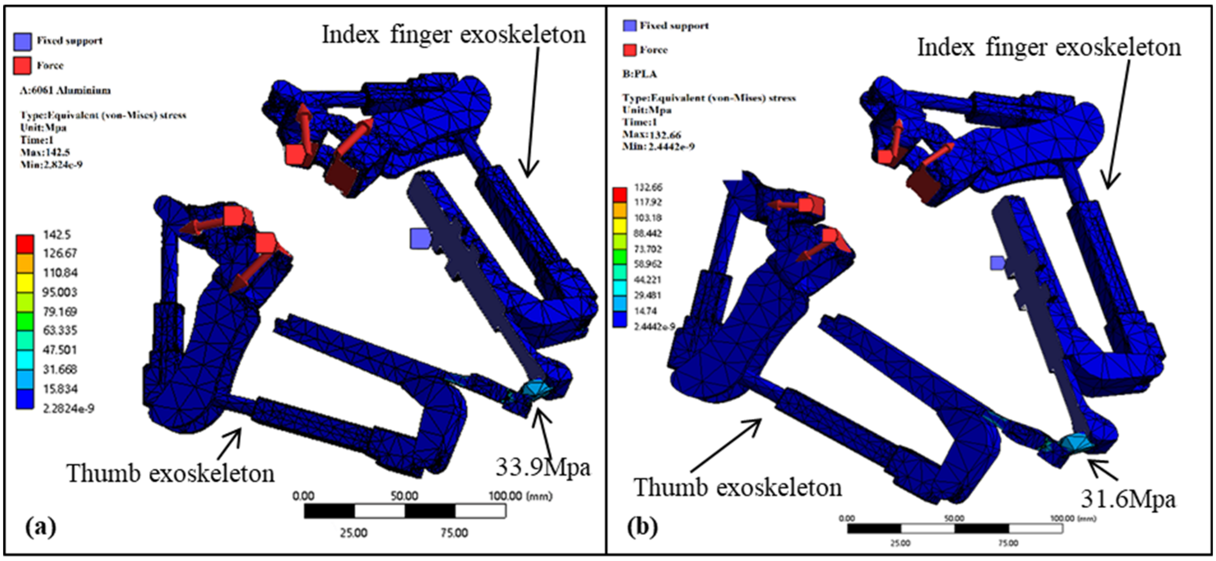

Figure S1. Stress evaluation for thumb exoskeleton and index finger exoskeleton. Index finger exoskeleton base serve as fix frame and with force (5N each) applied in the direction labelled with red color. The strength of the whole structure is also tested in real action. (a) For exoskeleton made by Aluminum 6061 the maximum stress is ~33.9 MPA, comparing with the material’s yielding stress; (b) For exoskeleton made by PLA material the maximum stress is ~31.5 MPA, comparing with the material’s yielding stress.

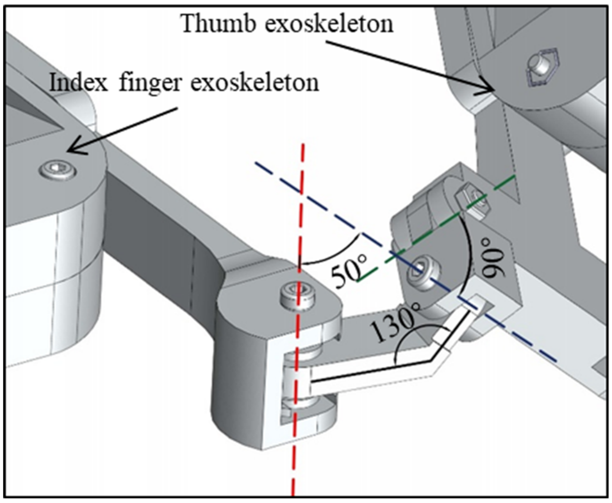

Figure S2. Schematic view of the 3 passive DoFs. These 3 DoFs can be fix for object grasp purpose.

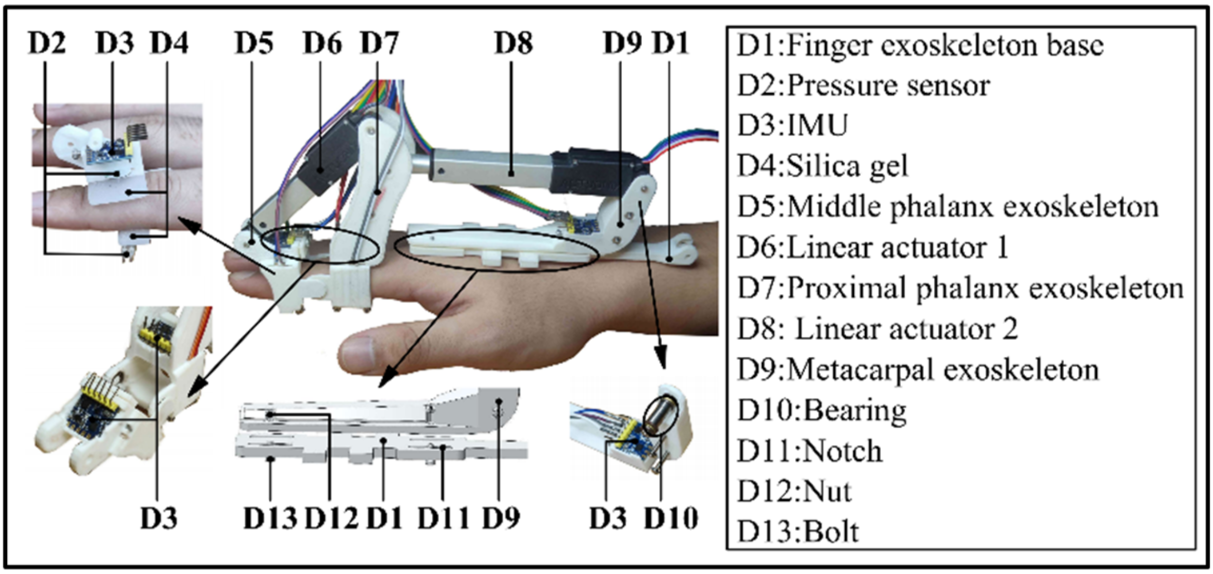

Figure S3. Components in the index finger exoskeleton. There 2 highlighted area in the finger exoskeleton, which illustrates the sensor locations and the sliding chute for length adjustment.

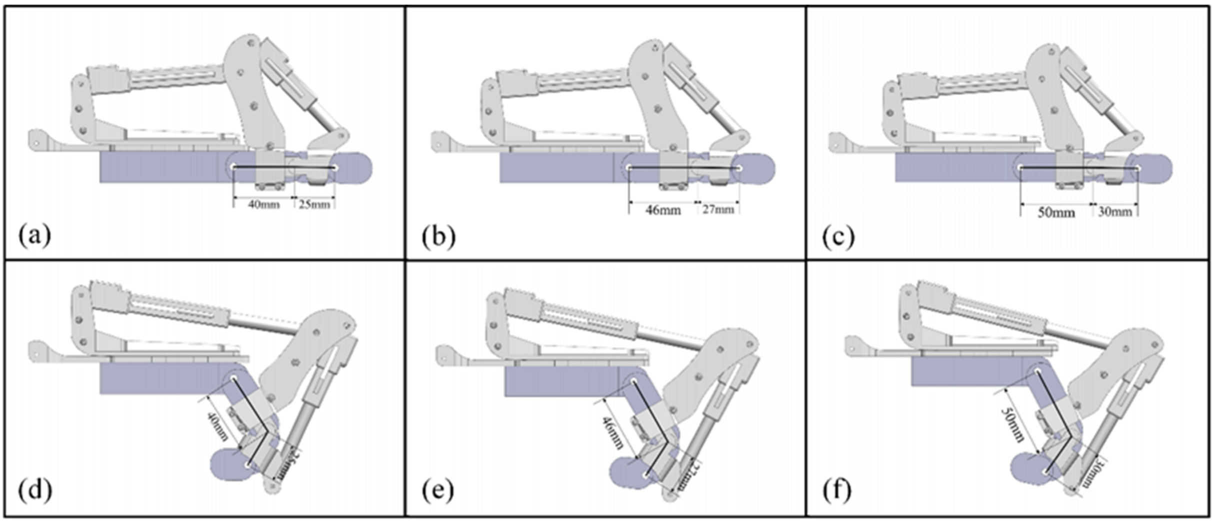

Figure S4. Hand exoskeleton worn by fingers of different phalanx lengths. (a-b) Length of proximal and intermediate phalanx are 40mm and 25mm respectively; (c-d) Length of proximal and intermediate phalanx are 46mm and 27mm respectively; (e-f) Length of proximal and intermediate phalanx are 50mm and 30mm respectively.

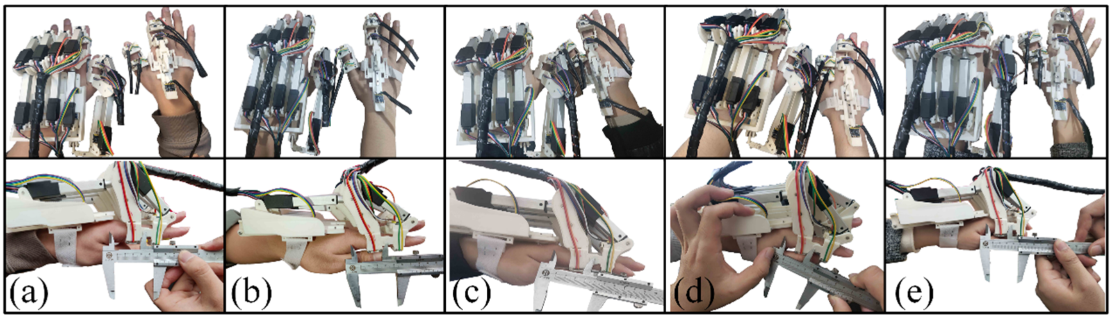

Figure S5. Index finger length of the 5 volunteers. (a) Proximal phalanx length, Middle phalanx length, and height of the volunteer are ~48mm, ~30mm and ~183cm respectively; (b) Proximal phalanx length, Middle phalanx length, and height of the volunteer are ~46mm, ~27mm and ~168cm respectively; (c) Proximal phalanx length, Middle phalanx length, and height of the volunteer are ~44mm, ~24mm and ~170cm respectively; (d) Proximal phalanx length, Middle phalanx length, and height of the volunteer are ~43mm, ~23mm and ~175cm respectively; (e) Proximal phalanx length, Middle phalanx length, and height of the volunteer are ~40mm, ~21mm and ~156cm respectively.

Table S1. The parameters for constructing Convolution Neural Network (CNN)

No	Layer Type	No. of Filters	Kernel/ Pool Size	Stride	Input Size	Output Size	Padding
1	Convolution 1	64	5	1	(None, 200,5)	(None,200,64)	Same
2	Batch-Normalization1						
3	Activation1				'Relu'		
4	Max-pooling1		2	1	(None,200,64)	(None,100,64)	Same
5	Convolution 2	128	5	1	(None,100,64)	(None,100,128)	Same
6	Batch-Normalization2						
7	Activation2				'Relu'		
8	Max-pooling2		2	1	(None,100,128)	(None,50,128)	Same
9	Convolution 3	256	5	1	(None,50,128)	(None,50,256)	Same
10	Batch-Normalization3						
11	Activation3				'Relu'		
12	Max-pooling3		2	1	(None,50,256)	(None,25,256)	Same
13	Dropout	0.2					
14	Flatten	(None,6400)					
15	Dense(128)	(None,128)					
16	Dense(6)	(None,6)					

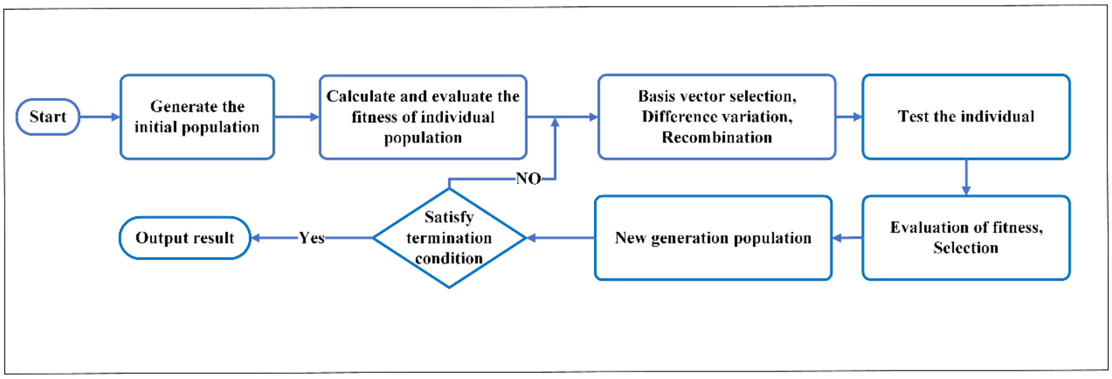

Figure S6. Flow chart of genetic algorithm (differential evolution algorithm)

Table S2. Genetic Algorithm setup for CNN model optimization

Differential evolution algorithm				
Parameter	Population quantity	Epoch	Scale factor F	Probability of crossover
	50	10	0.5	0.7
Optimization goal	Maximum: Accuracy of classification			

(Global)		
Decision variable	BatchSize	Value range: [30, 200]
	Epochs	Value range: [30, 500]
	LearningRate	Value range: [0.0005, 0.5]

Note: The average recognition accuracy of 10 times K-fold cross-validation is taken as the fitness function of population individuals, and the three hyperparameters (Learning-Rate, Batch-Size, Epoch) are taken as the decision variable.

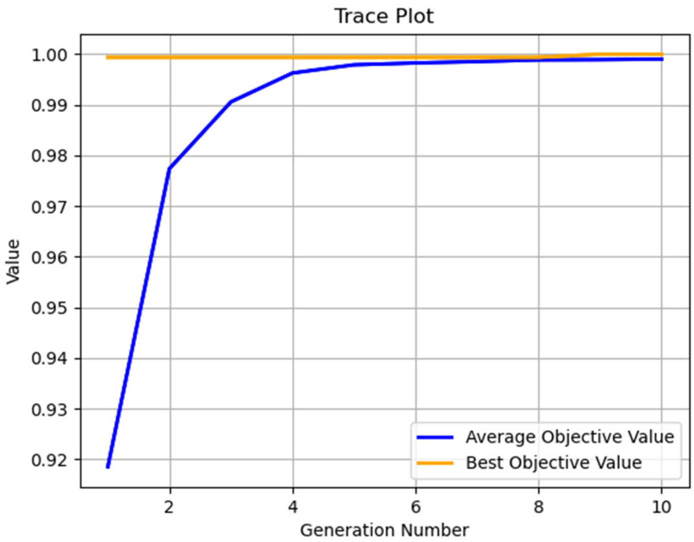

Figure S7. Results of genetic algorithm to optimize hyperparameters of CNN model

Table S3. The optimal value of hyperparameters in the CNN model

Generation	Optimal value	BatchSize	Epoch	LearingRate
10	1.0	136	9	0.38

Note: After 10 generations of population iteration, the optimal parameters of the model were obtained.

Table S4. Genetic Algorithm setup for SVM model optimization

Differential evolution algorithm				
Parameter	Population quantity	Epoch	Scale factor F	Probability of crossover
	50	50	0.5	0.7
Optimization goal (Global)	Maximum: Accuracy of classification			
	kernel functi	Value range: [0, 1] (Encoding: 0: “linear” 1: “rbf”)		

Decision variable	Penalty parameter	Value range: (0, 10]
	Gamma	Value range: (0, 10]

Note: The average recognition accuracy of SVM model on K-fold cross validation dataset is taken as the fitness function of individual population, and the three hyperparameter parameters mentioned above are taken as decision variables.

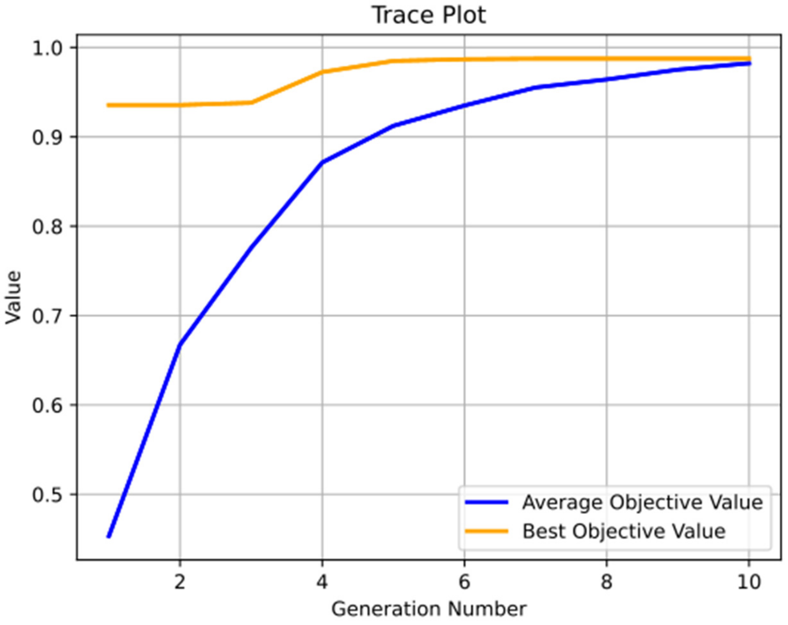

Figure S8. Results of genetic algorithm to optimize hyperparameters of SVM model

Table S5. The optimal value of hyperparameters in the SVM model

Generation	Optimal value	Kernel	C	Gamma
10	0.98	rbf	7	0.02

Note: After 10 generations of population iteration, the optimal parameters of the model were obtained.

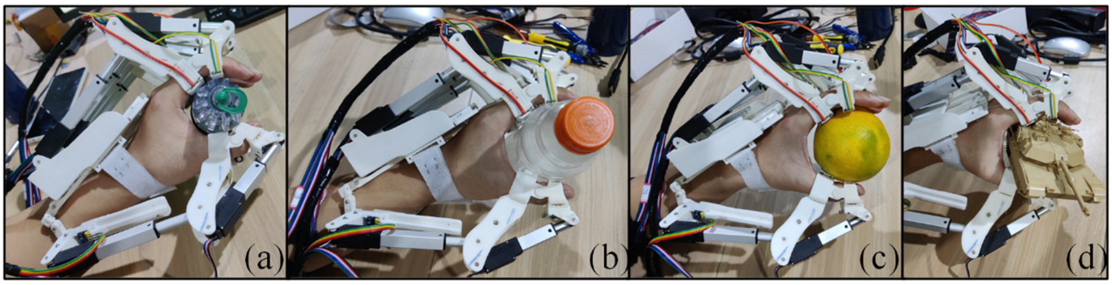

Figure S9. A demonstration of grasping objects with the passive joint setup illustrated in Figure S2. (a) A small toolbox with dimeter ~3.5cm; (b) water bottle with dimeter ~6cm; (c) Orange with dimeter ~6cm; A 1:35 M1A1 tank model.

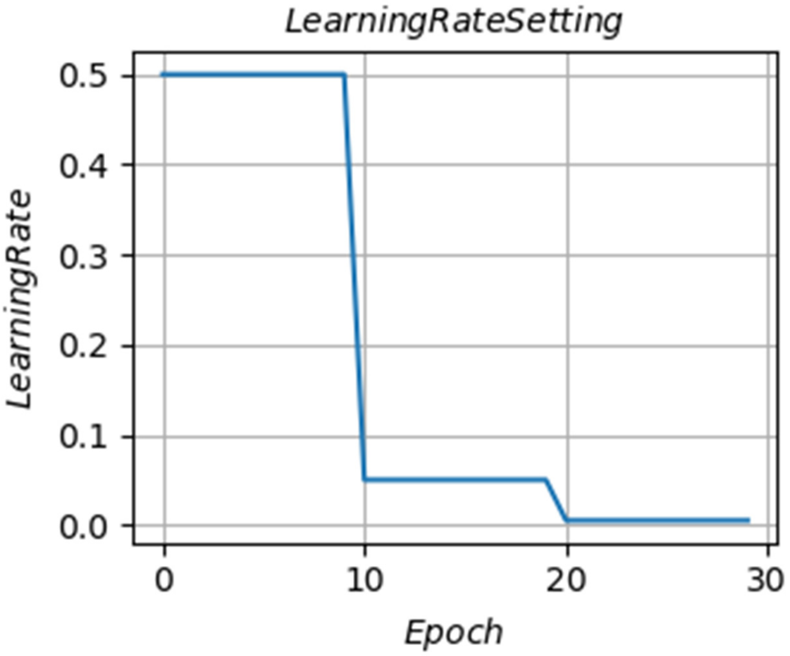

Figure S10. Curve of learning rate with epoch

In addition to model recognition accuracy (Table S1), model prediction time is also one important criterion to evaluate the quality of a model. As shown in Table S6 (Comparison of running time between CNN model and SVM model), CNN model and SVM model are respectively for a single data set prediction for 10 times, and the average time of the two models is obtained. Both models show fast response. However, as the size of data size increases to ~1200, time consumption maintains at ~0.45s, while it takes SVM several mins to finish the prediction.

Table S6. Prediction time using CNN model and SVM model

Time for prediction (s)	1	2	3	4	5	6	7	8	9	10	Average
SVM	0.0007	0.0006	0.0006	0.0006	0.0006	0.0006	0.0006	0.0006	0.0006	0.0006	0.0006
CNN	0.0662	0.0493	0.0401	0.0395	0.0400	0.0361	0.0393	0.0455	0.0523	0.0472	0.0462

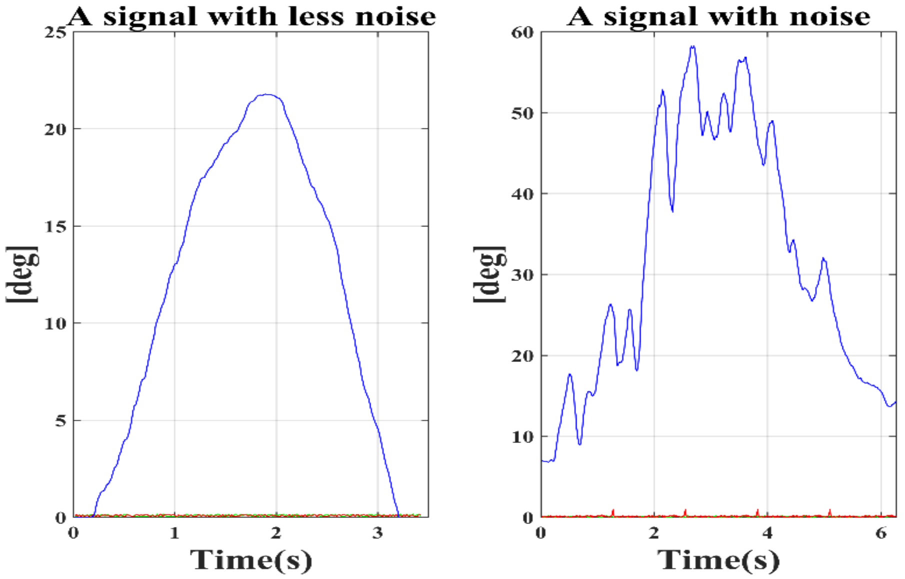

Figure S11. Comparison of identifiable signals with different levels of noise
